# The complete chloroplast genome of *Lilium nepalense* (Liliaceae)

**DOI:** 10.1080/23802359.2021.1872451

**Published:** 2021-02-11

**Authors:** Hongzhi Wu, Weiwei Bai, Shuilian He, Yan Zhao, Jingzhi Wu

**Affiliations:** Yunnan Agricultural University, Kunming, China

**Keywords:** *Lilium nepalense*, chloroplast genome, phylogenetic analysis

## Abstract

*Lilium nepalense* is a useful plant species not only for its showy flowers but also has high medicinal value. In this study, the whole chloroplast genome of *L. nepalense* was sequenced for the first time. The genome size of *L. nepalense*, was 152,956bp, with typical tetragonal structure: one large single copy (82,573 bp), one small single copy (17,527 bp), and a pair of inverted repeat regions (IRs, 26,428 bp). The overall GC content was 37.0%. The complete genome contained 131 genes, including 85 protein-coding genes, 38 tRNA genes, and 8 rRNA genes. Phylogenetic analysis showed that *L. nepalense* was a close relationship between *L. leucanthum* and *L. henryi*. Phylogenetic analysis placed *L. nepalense* under the family Liliaceae.

*Lilium nepalense* belongs to the perennial bulbous flower of Sect of Liliaceae. Its flowers are light yellow green, with green outside and purple spots inside. *Lilium nepalense* has a light fragrance, large number of flowers per plant, strong disease resistance, and adaptability, so with high ornamental value. It is one of the most promising wildflower resources in Yunnan. At present, there are no green flower varieties in lily cultivars (Guo et al. 2019). Therefore, *L. nepalense* is one of the potential parents to cultivate new varieties of green lily.

It is widely used in the Ayurvedic medicinal system, mostly as a tonic, and has been described as a stomachic, stimulant, and aromatic drug. The beautiful flowers are widely used for ornamental purposes. Due to the collection of large amounts of these plants, especially of the underground organs, with subsequent eradication of the natural populations, the natural habitats are rapidly decreasing (Wawrosch et al. 2001). At present, the fast propagation line of tissue culture of *L. nepalense* has been established successfully. The asexual propagation of *L. nepalense* has been carried out by tissue culture, which has enlarged the population on a large scale, not only fixed the excellent characters but also shortened the breeding cycle (Li et al. 2017). It provides the breeding technology guarantee for the protection of germplasm resources.

In this study, we determined the chloroplast genome sequence of *L. nepalense*, and discussed the genetic relationship among various species in Liliaceae.

The complete genomic DNA was extracted from fresh plant leaves and *L. rosthornii* was collected from Yuangyang County of Honghe prefecture in Yunnan province. Specimens were kept in Herbarium of Yunnan Agricultural University under the collection (No. 2020WHZ003). Total genomic DNA was isolated from fresh leaves using a DNeasy Plant Mini Kit (QIAGEN, Valencia, CA) according to the manufacturer’s instructions to construction chloroplast DNA libraries. Sequencing was carried out on an Illumina NovaSeq platform. The output was a 5 Gb raw data of 150 bp paired-end reads, further trimmed and assembled using SPAdes (Bankevich et al. 2012). Resultant clean reads were assembled using GetOrganelle pipeline (https://github.com/ Kinggerm/GetOrganelle). Annotations of chloroplast genome were conducted by the software Geneious (Kearse et al. 2012) and checked by comparison against the *L. leucanthum* complete chloroplast genome (GenBank accession number: KY748299).

The results of whole-genome sequencing showed that the size of chloroplast genome of *L. nepalense* (Genbank accession number: MW136391) was 152, 956 bp with a typical tetragonal structure: one large single copy (LSC, 82573 bp), one small single copy (SSC, 17527 bp), and two reverse repeats (IRS, 26428 bp). The total GC content was 37.0%, the GC content of LSC was 34.8%, and the GC content of SSC was 30.6%. A total of 131 genes were detected, including 85 protein-coding genes, 38 tRNA genes, and 8 rRNA genes. Nineteen gene are partially or completely duplicated, including seven PCG (rpl2; rpsl23; ycf2; ndhB; rps12; rps7; ycf1), eight tRNA (trnI-GAU, trnA-UGC, trnL-CAA, trnI-CAU, trnR-ACG, trnV-GAC, trnN-GUU, trnH-GUG), and all four rRNA (4.5S, 5S, 16S, and 23S rRNA). All the rRNA genes in the genome sequence were located in the repeat region.

Based on the chloroplast genome, 24 *Lilium* species and four outgroups were used to construct the phylogenetic tree of *Lilium*. According to the general time reversible substitution model in phyml 3.0, the maximum likelihood (ML) method was used to infer the phylogenomic relationships (Larkin et al. 2007; Guindon et al. 2010). The ML tree was constructed with 1000 bootstrap replicates using FastTree software (Liu et al. 2011). According to the result of the analysis, *L. nepalense* belongs to family Liliaceae and is associated with other *Lilium* species. The phylogenetic tree showed that 24 Lilium species were grouped into two branches, and *L. nepalense* was related to *L. leucanthum* and *L. henryi*. It has important medicinal and ornamental value and can provide a reference for other sect. In the meantime, it provides essential data for further study on the accurately identifying species, taxonomy, and phylogenetic resolution and evolution for the genus Lilium, and the available genome information could provide insight into conservation and exploitation efforts for this endangered ornamental and medicinal species (Figure 1).

**Figure 1.**
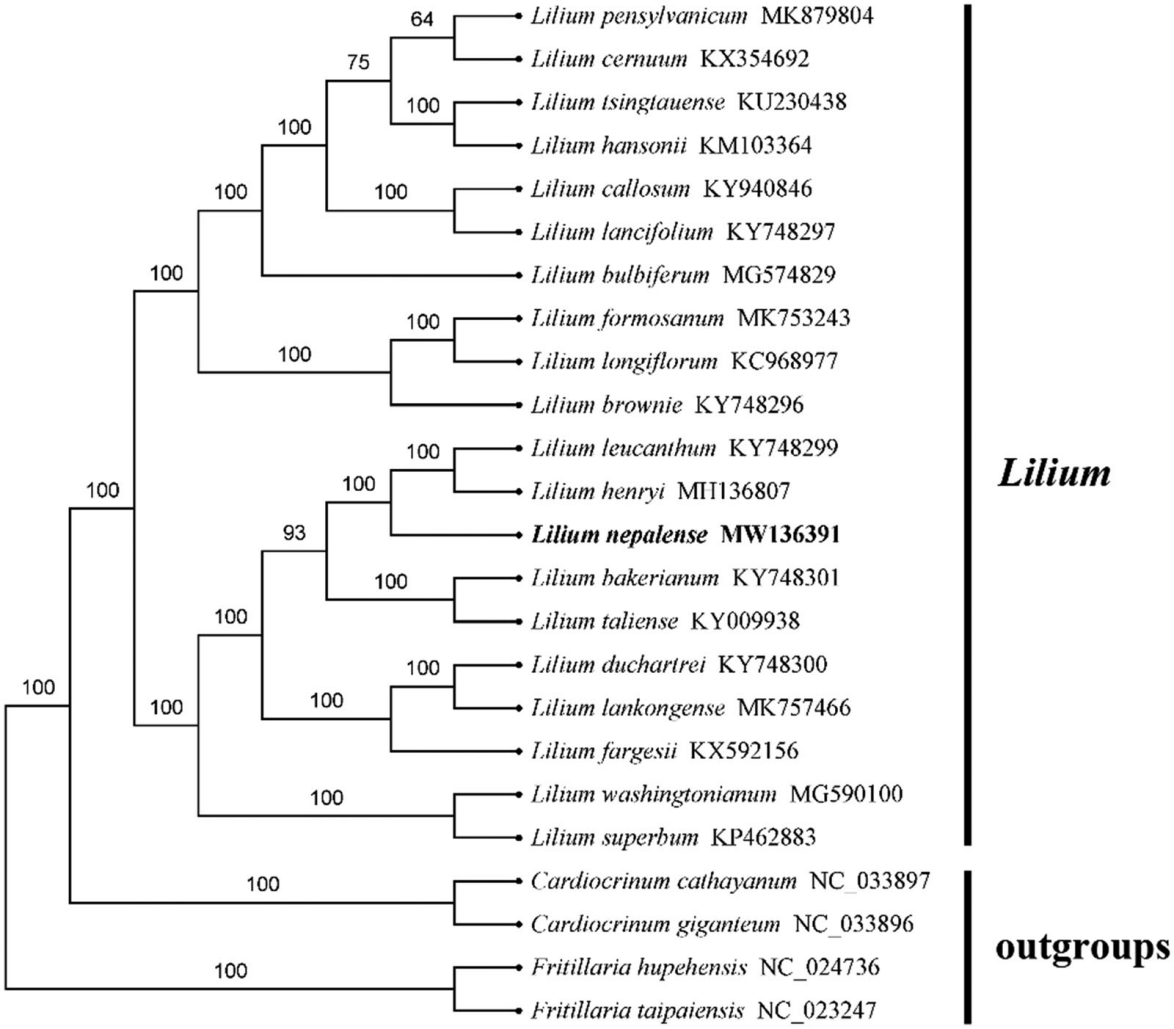
A phylogenetic tree of the Lilium species based on the completed chloroplast genomes of 24 species and 4 outgroup species. We downloaded all the other sequences from NCBI GenBank.

## Data Availability

The data that support the findings of this study are openly available in GenBank of NCBI at https://www.ncbi.nlm.nih.gov/, reference number MW136391.
